# Within-trial economic evaluation of diabetes-specific cognitive behaviour therapy in patients with type 2 diabetes and subthreshold depression

**DOI:** 10.1186/1471-2458-10-625

**Published:** 2010-10-19

**Authors:** Nadja Chernyak, Bernd Kulzer, Norbert Hermanns, Andreas Schmitt, Annika Gahr, Thomas Haak, Johannes Kruse, Christian Ohmann, Marsel Scheer, Guido Giani, Andrea Icks

**Affiliations:** 1 Department of Public Health, Center of Health and Society, Heinrich-Heine University Düsseldorf, Düsseldorf, Germany; 2 Institute of Biometrics and Epidemiology, German Diabetes Research Centre, Düsseldorf, Germany; 3 Diabetes Center Mergentheim, Bad Mergentheim, Germany; 4 Clinic for Psychosomatic and Psychotherapy, Universitäty Clinics Gießen/Marburg, Marburg, Germany; 5 Coordination Center for Clinical Trials, Heinrich-Heine University Düsseldorf, Düsseldorf, Germany

## Abstract

**Background:**

Despite the high prevalence of subthreshold depression in patients with type 2 diabetes, evidence on cost-effectiveness of different therapy options for these patients is currently lacking.

**Methods/Design:**

Within-trial economic evaluation of the diabetes-specific cognitive behaviour therapy for subthreshold depression. Patients with diabetes and subthreshold depression are randomly assigned to either 2 weeks of diabetes-specific cognitive behaviour group therapy (n = 104) or to standard diabetes education programme only (n = 104). Patients are followed for 12 months. During this period data on total health sector costs, patient costs and societal productivity costs are collected in addition to clinical data. Health related quality of life (the SF-36 and the EQ-5D) is measured at baseline, immediately after the intervention, at 6 and at 12 months after the intervention. Quality adjusted life years (QALYs), and cumulative costs will be estimated for each arm of the trial. Cost-effectiveness of the diabetes-specific cognitive behaviour group therapy will be analysed from the perspective of the German statutory health insurance and from the societal perspective. To this end, incremental cost-effectiveness ratio (ICER) in terms of cost per QALY gained will be calculated.

**Discussion:**

Some methodological issues of the described economic evaluation are discussed.

**Trial registration:**

The trial has been registered at the Clinical Trials Register (NCT01009138).

## Background

Depression is a highly prevalent disorder with a substantial impact on quality of life and societal cost [1,2]. This applies in particular to patients with diabetes, since depression has been shown to be more prevalent among these patients as compared to those without diabetes [3-5]. Previous research demonstrates that comorbid depression in patients with diabetes is associated with poor self care, i.e. adherence to medication, diet, exercise and smoking cessation [6-8], additive functional impairment and work disability [9], poorer glycaemic control [10], higher risk of microvascular and macrovascular complications [11,12], decreased quality of life [13], and higher mortality [14,15] as compared to patients with diabetes only. Many studies also found higher overall health care cost in depressed patients, not explained by higher mental health treatment costs alone. This association persisted even after adjustment for comorbid medical conditions [see e.g. [7,16-19]]. A US study [17] found 4.5-times higher total annual healthcare costs for Medicare patients with diabetes and comorbid depression than for diabetic patients without depression ($247,000,000 and $55,000,000, respectively; *P *< 0.0001 (cost adjusted to reflect August 2001 dollars).

Hence, the more effective depression treatment might not only improve health outcomes, but also reduce total health service utilization and therefore costs. Put differently, additional costs for improved depression treatment could be offset by reduction in other healthcare costs. In the IMPACT trial [20] for example, systematic depression treatment in older adults with diabetes had significant clinical benefit with no increase in overall healthcare costs. The Pathways Study [21] also found that over a 2-year period the increased costs associated with enhanced mental health treatment were offset by savings in total medical expenditures.

Cost-effectiveness of treatment options for depression has been mainly evaluated for major depression co-occurring with diabetes. However, there is a need to examine the cost-effectiveness of therapies for subthreshold depression as well, since there is evidence that about 20% of patients with diabetes have elevated depressive symptoms without meeting criteria for major clinical depression [4,5]. In what follows, a within-trial economic evaluation of a diabetes-specific cognitive behaviour therapy in patients with type 2 diabetes and subthreshold depression is outlined.

## Methods/Design

### Clinical trial

The clinical trial is carried out as randomised clinical trial comparing a diabetes-specific cognitive behaviour group therapy (DS-CBT) to a standard diabetes education programme (DEP). The trial is carried out in the in the Diabetes Centre Mergentheim, Germany, where about 6500 diabetic patients are treated annually. Approval for the study was granted by the local Medical Ethics Committee (Ethikkommission der Ärztekammer des Landes Baden-Württemberg). Primary endpoint of the clinical trial is the reduction of subthreshold depression at the 12 months follow up. Secondary variables are improvement of glycaemic control (HbA1c), health related quality of life (SF-36 and EQ-5D), diabetes related distress, and diabetes self-management. Assessments take place before the intervention (baseline), after the core intervention (at 2 weeks), and at 6 and 12 months of the trial.

### Sample size calculation

A remission in 65% and 40% of cases is assumed under the DS-CBT and DEP respectively. To detect a statistically significant difference with a power (1-β) of 90% (2-sided t-test, α = 0.05) 83 patients in each treatment group are required. Assuming a loss to follow-up of 20%, 104 patients in each arm of the trial are needed.

### Study sample

Patients with type 2 diabetes and elevated depressive symptoms (ADS > 22) [22], but without clinical depression according to Composite International Diagnostic Interview [23], 18 to 70 years of age who gave informed consent are included into the trial. Exclusion criteria comprise treatment with anti-depressant drugs, diagnosis of acute psychiatric illness and severe somatic illness.

### Interventions

Trials participants are randomly assigned either to the intervention group that receives the DS-CBT in addition to the standard diabetes education programme or to a control group that receives only standard diabetes education programme offered by the Diabetes Centre Mergentheim. The DS-CBT consists of 10 lessons of 45 minutes each, delivered in 5 sessions of 90 minutes in a group setting within a period of 2 weeks. Each DS-CBT group consists of minimum 3 and maximum 8 members. The DS-CBT is based on an evaluated German manual of cognitive behaviour therapy [24], which has been modified according to diabetes-specific topics (coping with self monitoring, self injecting of insulin, reactions of others to diabetes, coping with late complications, hypoglycaemia problems, keeping a healthy diet, barriers to lifestyle modification). Furthermore, 4 telephone contacts within the following year are offered as booster sessions in addition to the 5 sessions of the "core intervention". The diabetes education programme includes continuous education and group sessions to optimise the diabetes management as well as workshops and lessons with diabetes-specific topics.

### Within-trial economic evaluation

The objective of the economic evaluation, which is conducted alongside the trial, is to estimate the cost-effectiveness of the DS-CBT in terms of costs per quality adjusted year (QALY) from the perspective of the German statutory health insurance and from the societal perspective. To this end an incremental cost-effectiveness ratio (ICER) will be calculated, i.e. the ratio of the difference in costs between DS-CBT and DEP groups divided by the difference in QALYs gained in each group. Under statutory health insurance perspective on cost, ICER will be calculated using health sector costs only. Adopting the societal perspective, also patient costs and societal productivity costs will be added to the calculation of the ICER.

### Estimating effects of intervention in terms of QALYs

For the purposes of economic analysis, measures comparable across various interventions as well as across different disease areas are preferred. Most popular outcome measure for this purpose are the quality adjusted life-years (QALYs), which explicitly combine length and quality of life in a single measure, weighting survival (a set of health states) by utility scores. Utility weights reflect preferences for a particular health state and are measured on a scale from 0 to 1, where 0 and 1 represent death and full health, respectively [25]. Although the trial does not include a utility measurement as part of its protocol, it does include the SF-36 and the EQ-5D questionnaires measuring health related quality of life. Standardized algorithms exist to translate EQ-5D and SF-36 scores into utility weights suitable for calculation of QALYs [26-28].

More than 15 value sets are available for scoring the EQ-5D, based on rating scale and time trade-off (TTO) valuation derived from general population surveys in various countries (including the United Kingdom, Germany, and the United States) [28]. In this study the scoring function derived from a survey of the general population in Germany will be used to calculate utility weights from EQ-5D responses [29].

Brazier and colleagues reported work on deriving a reduced health status index from the SF-36 that they termed the SF-6D [30] and more recently, they have published an algorithm that allows the estimation of utility weights for all states of the SF-6D index [31]. Following this published algorithm, SF-36 scores observed in the trial will be converted to utility weights. Since the values underlying this algorithm were obtained in the United Kingdom, utilities derived from SF-36 scores will be only used to perform a sensitivity analysis.

The SF-36 and EQ-5D will be administered at baseline, immediately after the intervention, at 6 months and at 12 months after the intervention (see Table 1). Hence, a maximum of four possible observations for SF-36 and EQ-5D scores and derived utility weights will be available for each patient enrolled in the trial. QALYs will be calculated assuming linear interpolation between measurement points and calculating the area under the curve to give a number of QALY gained per patient over the trial period [25].

**Table 1:** Endpoints, measurement instruments and time of data collection

Endpoint	Questionnaire	Time of measurement			
		Baseline	2 weeks	6 months	12 months
QALYs	EQ-5D SF-36	**x**	**x**	**x**	**x**
Health sector costs	Health care utilisation and cost questionnaire	**x**		**x**	**x**
Patient costs	Health care utilisation and cost questionnaire	**x**		**x**	**x**
Productivity Costs	Health care utilisation and cost questionnaire	**x**		**x**	**x**

### Measurement of resource use

Resource use and costs directly associated with DS-CBT and DEP (e.g. staff time) will be derived from the therapy protocols. Information on the utilisation of other healthcare services will be obtained from trial participants by means of a cost questionnaire, which was developed for the study and incorporated into the case report files of the trial. The questionnaire is administered before the intervention (baseline), at 6, and 12 months of the trial and refers to the previous 6 months (see Table 1). The cost form includes structured no/yes questions on the utilisation of different medical services under the following categories: primary care visits, visits to emergency departments, visits to specialists, hospital stays, medication, and other therapies/paramedical care. If patients indicate that they received specific medical care over the past 6 months, they are asked to specify the volume: e.g. number of contacts with healthcare providers, number and length of hospitalizations, types and dosage of obtained medications. In the cost questionnaire patients are also asked to indicate whether health care services obtained by them were paid by the health insurance or self-paid, which makes an assessment of out-of-pocket expenses possible. Furthermore, the number of "days missed from work" will be registered with the cost questionnaire.

### Estimating costs

#### Health sector costs

To estimate costs from the statutory health insurance perspective, healthcare resource use due to interventions and other reported healthcare utilization (consultations, hospital days, etc.) will be multiplied by unit costs/prices. Currently, there are no German guidelines for costing in economic evaluations containing standard unit costs. Hence, healthcare resource use will be valued by unit costs/prices obtained from published sources and official statistics for Germany (e.g. charges and rates from administrative databases, pharmacy retail prices).

#### Patient costs

To estimate patient costs, reported consumption of healthcare services paid out of pocket will be multiplied by unit costs/prices available from official statistics and from providers.

#### Societal productivity costs

Days missed from work will be monetary valued according to the human capital approach [25].

### Statistical analysis of costs and effects

Mean total costs, health sector costs, patient costs and productivity costs as well as corresponding cost differences between the DS-CBT group and DEP group will be calculated. Sampling uncertainty (95% confidence intervals) will be estimated using bootstrap procedure because cost data are non-normally distributed.

Effect in terms of QALYs will be analysed using linear regression on type of intervention and - if necessary - on baseline utility score, which has been shown to be important for the unbiased assessment of mean QALY differences between treatment groups [32].

### Imputation of missing information on costs and effects

Data will be analysed according to the intention to treat principle. A multiple imputation approach based on propensity scoring will be used to account for missing information with regard to effects and costs. Baseline variables (e.g. age, gender, cost at baseline, etc.) will be entered into a logistic regression to predict the chance of a missing value [33,34]. Available data will be arranged into quintiles based on this predicted probability (propensity score) and a replacement value for missing data will be selected at random from the available data points within the same quintile. By choosing a value at random within the same quintile the principle of multiple imputations could be employed, whereby each missing value is replaced by m > 1 simulated values [35-37]. Each of *m *resulting data sets will be analysed as described above and combined to produce a single result that takes uncertainty in the imputation process into account.

### Determining cost-effectiveness

If a significant impact of DS CBT on both effects and costs is demonstrated, ICER will be estimated in terms of costs per QALY gained. ICER will be estimated for the total cost (health sector costs plus patient costs plus societal productivity costs) and for the health sector costs only (statutory health insurance perspective). The non-parametric bootstrap method will be employed to generate confidence intervals around the ICER estimates derived from the study sample [38,39]. Uncertainty surrounding the ICER will also be presented on the cost-effectiveness plane [40,41] and as the cost-effectiveness acceptability curve [42,43].

### Sensitivity analyses

Besides statistical uncertainty (sampling variation) with regard to costs and effects, every economic evaluation may contain some degree of data imprecision (e.g. resource costs/prices) and methodological controversy (e.g. derivation of utility weights, discount rate), which should be accounted for. To handle this type of uncertainty, sensitivity analysis is usually employed [25,44]. In the sensitivity analysis (uncertain) parameter(s) of the base-case analysis are varied to determine if changes in these parameters influence the results. Univariate sensitivity analyses will be performed by varying health service unit costs and utility weights (see Table 2). To appreciate the potential influence of missing responses and of the imputation method chosen, complete case analysis will be performed. We will report both the revised point estimates and revised confidence intervals for costs, effectiveness, and cost-effectiveness that result from the sensitivity analyses.

**Table 2:** Summary of planed sensitivity analyses

Parameter/methodological assumption	Base-case	Sensitivity analysis
Utility weights for QALYs	derived from the EQ-5D	derived from the SF-36

Unit costs/prices of resource use	data from published sources and official statistics for Germany	varied within a plausible range

Missing data	multiple imputation	complete case analysis

## Discussion

In the context of the trial, it might be argued that, if true randomisation is achieved, any differences in cost between treatment arms can be attributed to the study intervention [45]. Hence, data on utilisation of a broad range of health services will be collected in the trial. This approach allows measuring any changes in resource use related to the interventions being compared. On the other hand, however, it may complicate the detection of statistically significant difference in health service costs, since the latter have been shown to be highly variable and therefore to require larger overall sample sizes [45,46].

Information on healthcare utilisation other than DS-CBT and DEP sessions will be collected by self-report by means of the cost questionnaire. To our knowledge no standard and validated instruments for collecting resource use data in clinical trials are available in Germany. Hence, we developed a data collection instrument specifically for this trial. The questionnaire was pilot tested, but has not yet been validated against other data sources. Recall bias may potentially occur, since resource use will be measured over the previous 6 months. However, there is no conclusive evidence regarding whether a prospective (a cost diary) or a retrospective (a questionnaire) instrument should be better applied and regarding an appropriate recall interval [45]. Van den Brink et al. found that for the assessment of healthcare utilization in economic evaluations alongside clinical trials, a cost questionnaire may replace a cost diary for recall periods up to 6 months [47] and that such patients' self-reports are a valid source of data on days of hospitalization and out-patient visits, whereas costs of medication may be underestimated [48].

## Conclusions

Depression and depression symptoms co-occurring with type 2 diabetes are highly prevalent and associated with a wide range of adverse outcomes, including less effective self-care, more severe physical symptoms, greater functional impairment and disability as well as increased healthcare utilization and expenditure. However, there is a lack of evidence on cost-effectiveness of treatment options for subthreshold depression co-occurring with diabetes. The described trial-based economic evaluation will provide additional evidence on cost-effectiveness of the DS-CBT in this target group.

## Competing interests

The authors declare that they have no competing interests.

## Authors' contributions

AI and NC developed the design and methods for the economic evaluation alongside the clinical trial. NC wrote the manuscript. MS and GG gave support relating to the statistical analysis. BK is the coordinator of the clinical trial; NH and TH are principal investigators of the clinical trial; AS and AG are investigators of the clinical trial; JK advised on the design and methods of the clinical trial; and CO is responsible for data management. All co-authors read, edited, and approved the final manuscript. All authors participated in the work sufficiently to take public responsibility for respective parts of the paper.

## Pre-publication history

The pre-publication history for this paper can be accessed here:

http://www.biomedcentral.com/1471-2458/10/625/prepub
